# Tertiary and Quaternary Structure Organization in GMP Synthetases: Implications for Catalysis

**DOI:** 10.3390/biom12070871

**Published:** 2022-06-23

**Authors:** Lionel Ballut, Sébastien Violot, Frédéric Galisson, Isabelle R. Gonçalves, Juliette Martin, Santosh Shivakumaraswamy, Loïc Carrique, Hemalatha Balaram, Nushin Aghajari

**Affiliations:** 1 Molecular Microbiology and Structural Biochemistry, UMR5086 CNRS-University of Lyon1, 7 Passage du Vercors, CEDEX 07, F-69367 Lyon, France; lionel.ballut@ibcp.fr (L.B.); sebastien.violot@ibcp.fr (S.V.); frederic.galisson@ibcp.fr (F.G.); juliette.martin@ibcp.fr (J.M.); loic.carrique@strubi.ox.ac.uk (L.C.); 2 Microbiologie, Adaptation et Pathogénie, UMR5240 CNRS-University of Lyon1, F-69622 Villeurbanne, France; isabelle.goncalves@univ-lyon1.fr; 3 Molecular Biology and Genetics Unit, Jawaharlal Nehru Centre for Advanced Scientific Research, Jakkur, Bangalore 560064, India; porustg@gmail.com (S.S.); hb@jncasr.ac.in (H.B.)

**Keywords:** glutamine amidotransferase, GMP synthetase, *Plasmodium falciparum*, conformational changes, ammonia channel, allosteric regulation, signature motifs, dimeric interface, crystal structure, phylogenetic analysis

## Abstract

Glutamine amidotransferases, enzymes that transfer nitrogen from Gln to various cellular metabolites, are modular, with the amidotransferase (GATase) domain hydrolyzing Gln, generating ammonia and the acceptor domain catalyzing the addition of nitrogen onto its cognate substrate. GMP synthetase (GMPS), an enzyme in the de novo purine nucleotide biosynthetic pathway, is a glutamine amidotransferase that catalyzes the synthesis of GMP from XMP. The reaction involves activation of XMP though adenylation by ATP in the ATP pyrophosphatase (ATPPase) active site, followed by channeling and attack of NH_3_ generated in the GATase pocket. This complex chemistry entails co-ordination of activity across the active sites, allosteric activation of the GATase domain to modulate Gln hydrolysis and channeling of ammonia from the GATase to the acceptor active site. Functional GMPS dimers associate through the dimerization domain. The crystal structure of the Gln-bound complex of *Plasmodium falciparum* GMPS (*Pf*GMPS) for the first time revealed large-scale domain rotation to be associated with catalysis and leading to the juxtaposition of two otherwise spatially distal cysteinyl (C113/C337) residues. In this manuscript, we report on an unusual structural variation in the crystal structure of the C89A/C113A *Pf*GMPS double mutant, wherein a larger degree of domain rotation has led to the dissociation of the dimeric structure. Furthermore, we report a hitherto overlooked signature motif tightly related to catalysis.

## 1. Introduction

Purine metabolites are essential players in cellular processes and constitute one of the most abundant classes of molecules within mammalian cells. Besides being essential for DNA and RNA synthesis, they play a crucial role in providing cellular energy and intracellular signaling [1]. The synthesis of these highly important molecules, which are involved in energy transfer and metabolic regulation in both eukaryotes and prokaryotes, can follow two routes: either a de novo or a salvage pathway. Within this context, members of the glutamine amidotransferase enzyme family play a crucial role in the biosynthesis of nucleotides and amino acids. In humans, guanosine monophosphate synthase (GMP synthetase and/or GMPS) is required for the de novo biosynthesis of GMP and has, among others, been reported to play a major role in the invasion and tumorigenicity of cells from some human metastatic melanomas, with increased levels of GMPS in these latter compared to what is observed for primary melanomas [2]. Moreover, very recent results suggest that GMPS may be involved in the progression of cervical cancers, where it may be a marker of unfavorable prognosis of this cancer form [3]. In insects, GMPS has been documented to have a double function in that, besides being required for de novo GMP synthesis, it is involved in transcription control [4]. As concerns parasites for which the purine salvage pathway constitutes the only source of purine nucleotides, the enzyme has been shown to be essential for the viability and infectivity of the parasite *Trypanosoma brucei* [5]. For GMPS’ present in the de novo synthesis pathway in plants, the enzyme is used as a target for the design of herbicides [6], while their homologue in the encapsulated yeast *Cryptococcus neoformans* has been reported to be essential for virulence since, in its absence, the yeast cells become guanine auxotroph, leading to compromise in the production of key virulence factors [7]. Finally, in bacteria, inactivation of the GMPS (guaA) in *Clostridioides difficile* strain 630 led to cell death in minimal growth conditions, but not in a rich medium. Importantly, the capacity of a guaA mutant to colonize the mouse gut was significantly reduced. Together, these results demonstrate the importance of de novo GMP biosynthesis in *C. difficile* during infection [8].

GMP synthetase (EC 6.3.5.2) is a glutamine amidotransferase that catalyzes the final step of de novo GMP biosynthesis, whereby it converts xanthosine 5′-monophosphate (XMP) to guanosine 5′-monophosphate (GMP). GMPS is made up of two domains, one being a glutaminase (GATase) domain that catalyzes the hydrolysis of the amino acid glutamine to glutamate and ammonia. The generated ammonia is used by the ATP pyrophosphatase (ATPPase) domain to catalyze the amination of XMP to form the end product, GMP. It should be noted that whereas in eukaryotes and bacteria, these two domains are located on the same polypeptide chain, the catalytic reactions are carried out by two independent polypeptides in archaea. Hence, GMPS’ found in the first category are referred to as two-domain GMPS’, while those from the second category are named two-subunit GMPS’. Among the two-domain enzymes, substantial differences have been observed between the GMPS from the parasite *Plasmodium falciparum*, *Pf*GMPS, and the human homolog, thereby establishing these enzymes as attractive drug targets. An immediate example of these differences is that while the human enzyme has been reported to be a functional monomer [9], that of the parasite, together with other parasitic protozoa and prokaryotes, functions as dimers.

In an attempt to contribute to the detailed understanding of the mechanistic aspects of GMPS’, we have undertaken a series of in-depth studies of *Pf*GMPS to establish the relations between the structure, function and activity of these enzymes [10,11]. We, among others, have shown that upon binding of Gln to the GATase domain, this latter undergoes an 85° rotation and that this conformational change is required for channeling ammonia from this domain to the ATPPase domain in *Plasmodium falciparum* GMP synthetase. Upon this dramatic large-angle rotation, Cys113 (GATase domain) and Cys377 (ATPPase domain) are brought together, engendering the formation of a disulfide bridge in the crystal, as seen in the three-dimensional structure of *Pf*GMPS_C89A (mutation of one of the catalytic residues in the GATase domain) in complex with Gln. Moreover, we confirmed that the obligate functional dimer is formed via the ATPPase domain, as also observed in GMPS structures from prokaryotic organisms: *Escherichia coli* (PDB-ID 1GPM, [12]); *Thermus thermophilus* (2YWB, unpublished); *Coxiella burnetti* (3TQI, [13]); *Neisseria gonorrhoeae* (5TW7, unpublished); *Acinetobacter baummanii* (7SBC, unpublished).

Compared to their prokaryotic and parasitic counterparts, mammalian GMP synthetases display a 130 amino acid insert, and at least the *Homo sapiens* GMPS has been reported to be a functional monomer [9]. In order to get further insights into the functioning that distinguishes these glutamine amidotransferases, we performed detailed comparative studies of the existing dimer interfaces, but also compared these latter with the corresponding area in the human GMPS. Furthermore, we performed a phylogenetic analysis of GMP synthetases from various kingdoms of life to obtain a more fine-tuned understanding of the relevance of the extra-domain as found in mammalian GMPS’ compared to the homologues that lack this latter, including a number of eukaryotes. A lid-loop, essential for the key steps of the catalytic reaction and present in the ATPPase catalytic site, was also studied and compared with the homologous stretch in GMPS’ from other species. Finally, we have performed structural studies of the double mutant *Pf*GMPS_C89A_C113A, which sheds further light on domain rotation and its role in the functioning of the enzyme.

## 2. Materials and Methods

### 2.1. Cloning of PfGMPS and Mutagenesis

The three proteins studied herein were fused to an N-terminal 6His tag. The mutant *Pf*GMPS_C89A was constructed using pETPfGMPS [14] as a template. Mutant C113A was constructed by the single primer method by introducing a restriction site at the codon to be mutated, followed by knockout of the site to generate the desired mutation [10]. Native *P. falciparum* GMPS, *Pf*GMPSwt, was constructed as described earlier [14] from a native gene from PF10_0123 available in the *P. falciparum* genome database PlasmoDB (http://www.plasmodb.org (accessed on 1 January 2007)). The plasmid pETPfGMPS served as a template for the C113A mutation. Phusion DNA polymerase from Thermo Scientific was employed for the PCRs, and the resulting PCR products were digested with DpnI from New England Biolabs prior to transformation. The clones were verified by DNA sequencing.

### 2.2. Protein Purification

Protein expression and purification of the three enzymes studied herein were carried out as described earlier [10,14]. Briefly, *Pf*GMPSwt was expressed from the pQE30PfGMPS construct in *E. coli*, and the pETPfGMPS mutant constructs were transformed into BL21-CodonPlus(DE3)-RIL. Hereafter, colonies were inoculated into 5 mL Terrific Broth containing 100 mg × mL^−1^ ampicillin and 34 mg × mL^−1^ chloramphenicol and grown overnight at 37 °C. Inoculum (0.5%) of the overnight culture was then added to 50 mL Terrific Broth supplemented with antibiotics, grown at 37 °C for 6 H and thereafter used at 0.5% to inoculate 2.8 L of Terrific Broth supplemented with antibiotics. Induction with 0.5 mM IPTG (isopropyl-β-d-thiogalactopyranoside) was performed after culture growth to an OD_600_ of 0.6, and the growth continued for 18 H at 18 °C. Following harvesting and storage (−80 °C), the cell pellet was resuspended in 50 mM Tris-HCl, pH 7.4, 10% (*v*/*v*) glycerol, 0.1 mM DTT (dithiothreitol), and 0.1 mM PMSF (phenylmethanesulfonylfluoride), which will be referred to as Buffer A. The cell suspension was supplemented with 1 mM PMSF and sonicated on ice, and the cell lysate was centrifuged (30,500× *g* for 45 min). The supernatant of this latter was then loaded onto 1 mL HisTrap HP columns (GE Healthcare) using an ÄKTA HPLC (GE Healthcare). The column was washed with Buffer A containing 1 M NaCl, followed by step gradients of 20 to 100 mM imidazole in Buffer A. Proteins were eluted with Buffer A containing 1 M imidazole. Protein fractions were pooled and concentrated employing 30 kDa cut-off Amicon Ultra-15 centrifugal filter units from EMD Millipore. A final step of size-exclusion chromatography using a HiLoad 16/600 Superdex 200 pg column (GE Healthcare) was carried out in 20 mM Tris-HCl, pH 7.4, 10% (*v*/*v*) glycerol, 1 mM EDTA and 2 mM DTT, and the aliquots of the purified protein were flash frozen and stored at −80 °C. The yield was 5 mg of pure protein from 1 liter of culture.

### 2.3. Crystallization, Data Collection and Structure Determination

Crystallization condition screening was carried out at 292 K (vapor-diffusion in sitting drops), using commercially available crystallization kits. For screening, a Mosquito^®^ crystallization robot from STP Labtech was employed using two protein/crystallization agent ratios (200 nL + 200 nL and 300 nL + 100 nL drops equilibrated against 70 µL in MRC Crystallization Plates (Molecular Dimensions)). The mutant protein *Pf*GMPS_C89A_C113A was concentrated to 13 mg × mL^−1^ in 50 mM Tris-HCl pH 8.0, 100 mM NaCl buffer. Crystals grew in a mixture of 16% polyethylene glycol (PEG) 3350, 0.06 M sodium citrate (pH 2.3) and 0.04 M Bis-Tris propane (pH 9.7). Prior to data collection, the crystal was briefly soaked in a precipitant solution to which 15% ethylene glycol (final concentration) had been added and flash frozen in liquid nitrogen.

X-ray diffraction data were collected on a single crystal (ID30A-3—ESRF, Grenoble, France) at a wavelength of 0.87290Å. Data processing was done using programs from the XDS package [15]. Phases and experimental electron density maps were calculated after a molecular replacement search using the *Pf*GMPSwt three-dimensional structures [10] of the C89A single mutant in complex with glutamine (PDB-ID 4WIO), the native structure of the isolated GATase domain (PDB-ID 4WIN) and the ATPPase domain extracted from the full-length structure of the native enzyme (PDB-ID 4WIM) as search models with the PHASER [16] program. The initial model was built using Phenix AutoBuild [17]. Hereafter, alternating cycles of model building and refinement were done employing Coot [18] and REFMAC [19]/Phenix [20].

### 2.4. Small Angle X-ray Scattering

For SAXS data collection, the samples were concentrated to ~12 mg × mL^−1^. Data were collected on beamline BM29 (ESRF, Grenoble, France) at a wavelength of 0.9919 Å. The data were analyzed using the ATSAS program package [21]. Ab initio models (50 in total) of *Pf*GMPS_C89A_C113A were generated from the experimental data. After averaging the 50 generated models, they were filtered to generate the final model.

### 2.5. Characterization of GATase Domain Rotation

Two dynamic domains, corresponding to the GATase and ATPPase structural domains, were identified by the program DynDom [22] from pdb files of *Pf*GMPSwt, *Pf*GMPS_C89A/Gln and *Pf*GMPS_C89A_C113A (monomer of each structure). Hereafter, UCSF ChimeraX v1.3 [23] was used to superimpose *Pf*GMPSwt or *Pf*GMPS_C89A/Gln onto *Pf*GMPS_C89A_C113A, holding the ATPPase domain fixed. After superposing the ATPPase domains, the change in orientation of the GATase domains was established using ChimeraX [23], which gave the rotation angle and translation along the rotation axis.

### 2.6. Phylogenetic Data Analysis

Homologs were searched against the UniProtKB database using the program BLASTP [24] and *Ec*GMPS and *Hs*GMPS as query sequences (Supplementary Table S1). To ensure the correct representation of the different taxonomic groups, a total of 42 BLASTP searches were run on the NCBI website. Herein, each search set was limited to one of the taxonomic groups, as represented in Supplementary Table S1. All sequences that aligned at least 80% of the length of the two and/or three canonical domains (GATase, ATPPase, extra-domain) of *Ec*GMPS/*Hs*GMPS sequences were kept for multiple sequence alignment. Hereafter, sequences were clustered with CD-HIT to maintain a significant number of sequences for each group. To further enrich the phylogenetic tree, sequences were manually added: 20 well-known model organisms taken from the Uniprot database, 11 from prokaryotes [25], and the sequences corresponding to the existing three-dimensional structures in the protein databank at Rutgers [26]. Multiple sequence alignment was done with the MAFFT program [27] and conserved blocks were selected employing BMGE 1.12 [28]. The BLOSUM62 [29] matrix was used in both programs. 408, 158 and 252 sites were kept for the full-length protein, the GAT domain and the ATPPase domain, respectively, for further analysis after character trimming performed by BMGE [28]. Phylogenetic analyses were performed with the LG model and gamma correction, employing a maximum-likelihood approach with PhyML [30]. 100 bootstrap replicates were performed. The phylogenetic tree was generated and rendered with iTOL software [31].

### 2.7. Bioinformatic Analysis

For the 90 selected sequences used for phylogeny, sequence analyses were done using the Bioinformatics Toolkit resource at https://toolkit.tuebingen.mpg.de (accessed on 4 April 2022) [32,33]. Clustal Omega v1.2.1 [34] was employed for alignment of the protein sequences, and secondary structure predictions of aligned sequences were performed by PSIPRED [35] employing the Ali2D Tool of the Bioinformatics Toolkit resource. Sequence logos were displayed using the WebLogo 3 resource (http://weblogo.threeplusone.com (accessed on 15 April 2022)) [36].

### 2.8. Figure Rendering

Figures with three-dimensional structure representations were rendered using PyMOL [37] and UCSF ChimeraX v1.3 [23].

## 3. Results

Although the first structural details of a GMPS were published in 1994 [12] and since then 12 more structures of full-length GMPS’ have been added to the pdb databank, some aspects of the molecular mechanism of catalysis by this enzyme still remain elusive. In our previous studies of a *Pf*GMPS mutant in which one of the catalytic residues in the GATase domain, Cys89, was mutated to an alanine, the three-dimensional structure in complex with the substrate Gln revealed that this domain was rotated by 85° as compared to the wild-type enzyme [10]. Here, we present novel findings that add further insight into the functioning of this enzyme with a structure of a variant in which not only the catalytic Cys89 was mutated, but also Cys113, which was earlier shown to form a disulfide bridge with Cys377 in the *Pf*GMPS_C89A/Gln complex.

### 3.1. Crystal- and Solution Structures

The *Pf*GMPS_C89A/C113A mutant enzyme was crystallized, and the crystals of this double mutant belonged to the monoclinic space group *C*2. The crystal structure of this mutant solved by molecular replacement was determined to a 2.8 Å resolution. The asymmetric unit contains 1 molecule, and all residues have been modeled with the exception of flexible regions with no electron density (residues 234–237, 342–343, 390–396, 487–495 and 521–538). The data collection and refinement statistics are given in Table 1.

**Figure 1:**
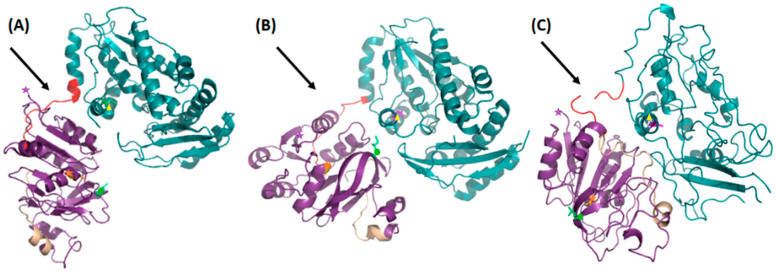
Crystal structures of (**A**) *Pf*GMPSwt, (**B**) *Pf*GMPS_C89A in complex with Gln and (**C**) *Pf*GMPS_C89A_C113A. GATase domains are colored in purple, the linker regions in red, the ATPPase domains in teal and the unique (parasite enzyme) insertion including residues 14–159 in wheat. In order to visualize the changes in orientation between the domains with the ATPPase domain held fixed, the N-terminus has been labeled with a purple star, C89 (catalytic residue in the GATase domain) with an orange star, C113 (residue forming a disulfide bridge with C377 as observed in the crystal structure of *Pf*GMPS_C89A in complex with Gln) with a green dot and D371 (catalytic residue in the ATPPase domain) with a yellow triangle, respectively. When comparing the three ATPPase domains, it was noticed that this latter is completely destructured in the double mutant described herein (**C**). The linker is positioned in front of the GATase domain in the native enzyme (**A**), behind the GATase domain in the single mutant enzyme (**B**) and is unstructured in the double mutant enzyme (**C**).

When comparing the crystal structure of this double mutant with the crystal structures of the native enzyme and with that of the C89A/Gln mutant complex, substantial conformational changes were observed. The GATase domain of the double mutant (*Pf*GMPS_C89A_C113A) is rotated by ~170° and translated ~1.5 Å compared to the native structure and when holding the ATPPase domain fixed. If compared with the structure of the single mutant, the GATase domain is rotated by ~120° and translated ~4 Å, again when holding the ATPPase domain fixed (Figure 1).

The electron density of this double mutant revealed a crystal structure that is not only more collapsed than the wild-type and single mutant enzymes described earlier but also significantly less structured, resulting in bad geometry, as can be observed from the Ramachandran parameters. Destructuring is particularly important in the ATPPase domain, and to our surprise, the observed structure of this double mutant is a monomeric enzyme. Moreover, the collapsed nature of the structure has resulted in many contacts being very close. Here, it should be mentioned that numerous attempts to grow better crystals of this mutant failed.

**Figure 2:**
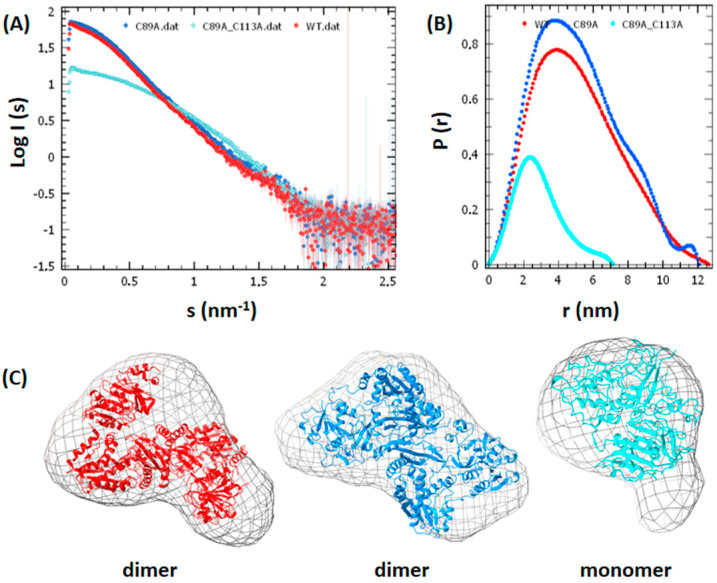
Solution structures of *Pf*GMPSwt, *Pf*GMPS_C89A and *Pf*GMPS_C89A_C113A as determined by Small Angle X-ray Scattering. The (**A**) radially scattered X-ray intensity (I) as a function of the scattering angle (s), with *Pf*GMPSwt dimer (red), *Pf*GMPS_C89A dimer (blue) and *Pf*GMPS_C89A_C113A monomer (cyan). (**B**) Pair-distance distribution functions P(r) with the same color codes as for (**A**). (**C**) Crystal structures with the same color coding as above the respective variants superimposed onto the averaged ab initio shape reconstructed with DAMMIF [39].

The monomeric nature of the double mutant in solution was confirmed by Small Angle X-ray Scattering studies (Figure 2), as opposed to the solution dimers observed earlier for the *Pf*GMPSwt and *Pf*GMPS_C89A enzymes [10] and confirmed herein.

Indeed, *Pf*GMPS_C89A_C113A lacks Gln-dependent GMPS activity due to the mutation of the catalytic cysteine and shows a 6.7-fold decrease in *K*_m_ for NH_4_Cl compared with the wild-type enzyme and a 2.9-fold decrease compared with the single mutant *Pf*GMPS_C89A. With *k*_cat_ values being almost similar, *Pf*GMPS_C89A_C113A had the highest *k*_cat_/*K*_m_ value for ammonia-dependent GMP formation. Nevertheless, the conservation of enzyme activity shows that this double mutant may also adopt a dimeric form. However, the structure of *Pf*GMPS_C89A_C113A shows that the double mutant at the extreme rotation of the GATase domain transitions from a dimer to a monomer conformation. Careful inspection of the three-dimensional structure of the *Pf*GMPS_C89A_C113A mutant suggests that this destructuring is a result of an aberrant rotation of the GATase domain in conjunction with a unique insertion (Asp141-Ile159; Figure 1) in this same domain being specific to the parasite enzyme [38]. Taken together, these issues engender the destabilization of the dimer interface, which has been shown to be essential for enzyme activity. Unequivocally, the double mutant is no longer able to form this disulfide bridge, resulting in an enzyme that, in principle, can continue its rotation. Here, it should be noted that Cys377 is only found in Plasmodium and Cys113 only in *P. falciparum*, *P. vivax* and *P. knowlesi*. Interestingly, Cys377 is part of the so-called lid-loop, which precedes the helix that holds Asp371 and Glu374 (catalytic residues of the ATPPase domain) and is, as evident from our earlier mutagenesis studies, a key residue in the AMP-XMP intermediate formation. The formation of a disulfide bridge in this position has only been observed in the *Pf*GMPS_C89A/Gln crystal structure among the GMPS structures solved to date.

It should be noted that to measure the rotation of the GATase domain in solution, a FRET-based assay was conceived. For this experiment, Trp167 from the GATase domain was chosen as the FRET donor, and Cys377 from the ATPPase domain was covalently labeled with 5-(2-((2-iodoacetyl)amino)ethylamino)naphthalene-1-sulfonic acid “IAEDANS” as the FRET (Förster Resonance Energy Transfer) acceptor. Inspection of the crystal structures of native *Pf*GMPSwt and the rotated *Pf*GMPS_C89A indicates that upon the 85° rotation of the GATase domain, the distance between these two residues is reduced by approximately 14 Å, from ~19.5 Å to ~5.5 Å. To ensure that only the above-mentioned Trp and Cys were labeled, the second tryptophan and the 10 remaining cysteines in the enzyme were mutated to phenylalanine and alanines, respectively. Unfortunately, this resulted in a mutated enzyme that was insoluble.

Altogether, one may conclude that whereas this double mutant (*Pf*GMPS_C89A_C113A) can adopt a dimeric form, as concluded from the conservation of the enzyme activity, it is also able to transit to a monomer conformation at the extreme rotation of the GATase domain, as confirmed by the crystal structure and Small Angle X-ray Scattering studies.

### 3.2. Comparative Studies of Dimerization Interfaces

**Figure 3:**
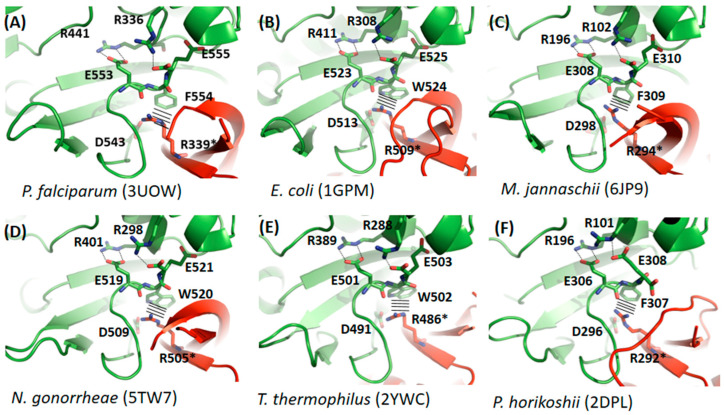
Conservation of amino acid residues involved in interactions with XMP in the ATPPase domain and the stabilization of the dimer interface. Lines indicate π—cation interactions. For GMPS from (**A**) *P. falciparum*, (**B**) *E. coli,* (**C**) *M. jannaschii*, (**D**) *N. gonorrhea*, (**E**) *T. thermophilus* and (**F**) *P. horikoshii* residues from monomer A are shown in green and monomer B in red with the interacting arginines marked by an asterisk.

Considering that all our recent (described above) and earlier [10,11] observations of *Pf*GMPSwt indicate that in two-domain dimer GMPS’, the dimer interface is essential for forming a scaffold allowing interaction with the substrate XMP, we decided to elaborate on this issue. A close-up on the GMPS dimer interfaces, allowing a detailed inspection of the interacting residues in this area (Figure 3 and Supplementary Figure S1), indicates that these interactions are conferred by the C-terminus of the enzyme. Comparison of the dimer interfaces of GMPS’ of known structure reveals that these interactions are partly highly conserved among the different species (Figure 3) and involve two arginines, two glutamic acids, an aspartic acid and an aromatic residue from one monomer and an arginine from a second monomer. Some of these residues (Glu553, Phe554 and Glu555, *Pf*GMPS numbering) are involved in the highly conserved C-terminal signature motif KPPXTXE(F/W)X. Within this motif, Glu553 has been determined to be critical for XMP binding (mutation to Leu), whereas for Glu555 the Gln and NH_4_Cl dependent GMP formation by the *Pf*GMPS_E555L mutant was only marginally lower than what was observed for the wild-type enzyme [11].

A second part of the interface is less conserved (Supplementary Figure S1A–F) and rather seems to be specific for the organism as *eg*. *T. thermophilus* GMPS which is stabilized by a salt bridge, which is a frequent stabilizing factor in proteins from thermophilic species.

**Figure 4:**
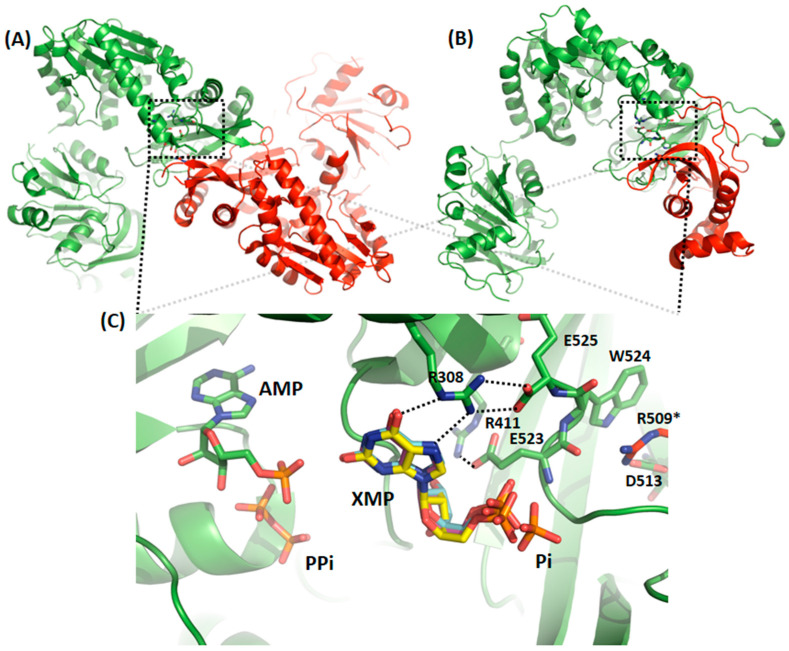
Overall three-dimensional structure of GMP Synthetases from a two-domain dimeric enzyme as exemplified by (**A**) *P. falciparum* GMPS dimer with one monomer colored in green and the other in red, versus that of a two-domain monomeric enzyme as exemplified by the (**B**) *H. sapiens* GMPS monomer in which the extra-domain is colored in red. The rectangle in the stippled lines delineates the substrate-binding site in the ATPPase domain in the two structures. (**C**) Close-up on the ATPPase active site in structures with bound substrate (XMP in *Hs*GMPS—PDB 2VXO, yellow; in *Tt*GMPS—PDB 2YWC, light blue; in *Pf*GMPS—PDB 3UOW, pink and AMP, PP_i_ and P_i_ in *Ec*GMPS, green and orange). To simplify, only protein residues (in green) of the *E. coli* enzyme are shown in this figure. As can be seen from the interaction scheme, arginine 308 (see numbering correspondence for the other GMPS’ in Figure 3) interacts with the C-terminal and together with the remaining residues allow the interaction with XMP and the stabilization of the interface corresponding to the so-called dimerization interface.

For mammalian GMPS’, as exemplified by *Hs*GMPS, which is a monomeric enzyme, the overall fold differs from that seen for functional dimeric enzymes as eg. *Pf*GMPS, in that *Hs*GMPS displays an extra-domain (known as the D1 sub-domain) made up of 130 amino acid residues. This subdomain, which is present in mammalian GMPS’, is involved in substrate binding but also in dimerization, as seen in the crystal structure of *Hs*GMPS, which turned out to be a dimer in the crystal [40]. This extra-domain insertion is located in the ATPPase domain (Figure 4), and the orientation of the D1 subdomain and the subsequent C-terminal part (referred to as subdomain D2 by Welin and colleagues [40]), mimics the dimer interface as formed by ATPPase domains of two monomers seen in functional dimers such as that of the *P. falciparum* parasite (Supplementary Figure S2). Moreover, as also indicated by the authors, when comparing subdomains D1 and D2 within the *Hs*GMPS, they display a similar polypeptide fold when the two sub-structures are superimposed. It should be highlighted that the aforementioned “subsequent” C-terminal part is present in all GMP synthetases (Figure 3 and Figure 4) and includes, among others, the highly conserved C-terminal signature motif, as mentioned above. As seen in Supplementary Figure S2 and Figure 3, the residues involved in the interactions with the XMP substrate are intriguingly highly conserved, despite the fact that part of the residues stem from the extra-domain in the human enzyme instead of a second molecule, as is the case for the dimeric GMPS’. To summarize, when comparing the interactions seen in GMPS’ of known three-dimensional structures, in one part of the region corresponding to the dimer interface in dimers as exemplified by *Pf*GMPS and the region mimicking this same region in monomers as *eg*. *Hs*GMPS conservation of amino acid residues involved in interactions with XMP and in the stabilization of the dimer interface are observed. As opposed hereto, the second part of the interface seems more specific to the organism and shows a lower degree of conservation.

### 3.3. Phylogenetic Analyses

Based on these considerations and in order to get a better understanding of the evolution of these multi-domain architectural proteins, for which two different quaternary structures constitute the physiologically active forms, we decided to look at the phylogenetic relationships between these enzymes.

As a means to start with representative samples of these two groups, the GMPS protein sequence from *E. coli* and/or *H. sapiens* was used in a NCBI blastp search against 42 different proteomes belonging to Bacteria, Archaea and Eukaryotes. The retrieved sequences were then clustered to maintain a representative number of sequences for each group. Nineteen sequences of model organisms were then added along with sequences of bacteria used in an earlier phylogenetic analysis [25]. Different trees were constructed either using the complete protein sequences or by limiting the analysis to a single domain (i.e., GATase or ATPPase) and similar results were obtained.

**Figure 5:**
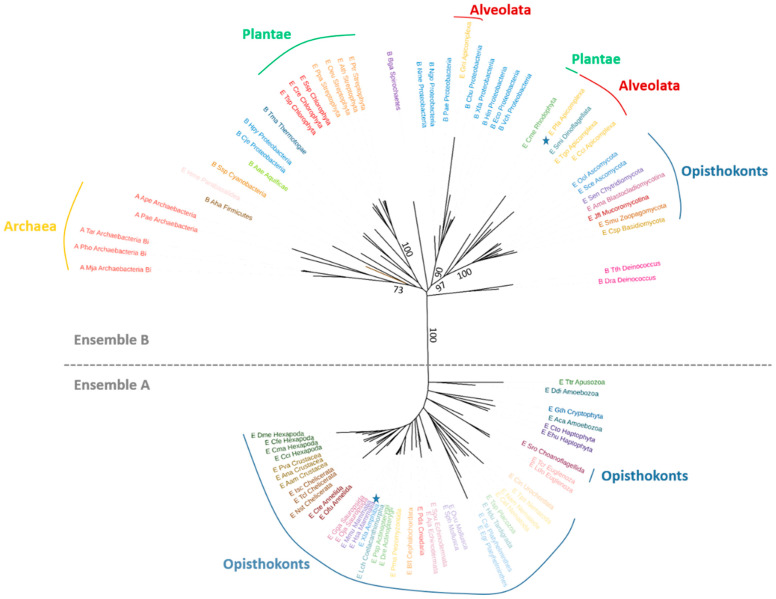
Phylogenetic tree of full-length GMPS enzymes. A ML phylogeny based on 408 amino acid alignment positions of 87 sequences was constructed with PhyML [30] using GMPS’ from eukaryotes, archaea and bacteria. *Pf*GMPS and *Hs*GMPS have been labeled with stars. A clear separation between a group of eukaryotic proteins (ensemble A) and a group of both prokaryotic and eukaryotic proteins (ensemble B) can be observed.

For each tree, a clear separation occurred between a group (ensemble A) of eukaryotic proteins and a group (ensemble B) of both prokaryotic and eukaryotic proteins (Figure 5 and Supplementary Figures S3 and S4). Eukaryotic organisms found in ensemble A belong to the Opisthokonta, Apusozoan, Amoebozoa, Euglenozoan, Haptophyta and Cryptophyta. Ensemble A gathers protein sequences displaying (in the ATPPase domain) a conserved “extra-sequence“ (residues 450-578 in *H. sapiens* GMPS corresponding to the dimerization domain D1) absent in the protein sequences found in ensemble B.

Based on our above-standing comparative analysis of the three-dimensional structures of GMPS’ available in the Protein Data Bank RCSB [26], we observed that the site mimicking the dimer interface is both issued from the extra-sequence and another part of the protein sequence, which is present in all GMP Synthetases (Figure 4). Prediction of the expected secondary structures of this extra-domain for each of the studied sequences from ensemble A indicates conserved β-strands and α-helices (Figure S5). In accordance with the monophyly of ensemble A proteins in the phylogeny, the complex structural rearrangements, which end up by the acquisition of the extra-domain existing in these proteins, suggest that they have certainly occurred only once during evolution in the ancestor of the ensemble A proteins.

In ensemble B, the extra-sequence is systematically absent, suggesting that the active form of all these proteins displays a dimeric organization. Organisms for which the GMPS three-dimensional structures have been solved and which form dimers (*E. coli*, 1GPM; *T. thermophilus*, 2YWC; *P. horikoshii*, 2DPL; *C. burnetti*, 3TQI; *P. falciparum* 3UOW and 4WIM; *N. gonorrhea*, 5TW7; *M. jannashii*, 6JP9; *A. baumannii*, 7SBC) are all present in this second group supporting this hypothesis. Ensemble B is constituted by bacteria, archae and eukaryotes. Among the eukaryotes, we find Plantae (Streptophyta, Chlorophyta and Rhodophyta), Fungi, Parabasalidea and Alveolata (Dinoflagellata and Apicomplexa). No species possessing either type of protein was found.

The presence of the extra-domain in most Opistokonts, but also in Apusozoan and Amoebozoa GMP Synthetases, suggests that it was present in the ancestral Opisthokonta proteins. Surprisingly, among the Opisthokonta, fungal GMPS’ do not possess the extra domain. Fungal sequences form a monophyletic group in ensemble B, suggesting the replacement of an ancestral Opistokhonta version of the gene (with the extra-sequence) by a laterally transferred gene. Such a GMPS-encoding lateral gene transfer (LGT) was suggested in an earlier study [25].

### 3.4. Comparative Studies of the Catalytic Loop Region in the ATPPase Domain

**Figure 6:**
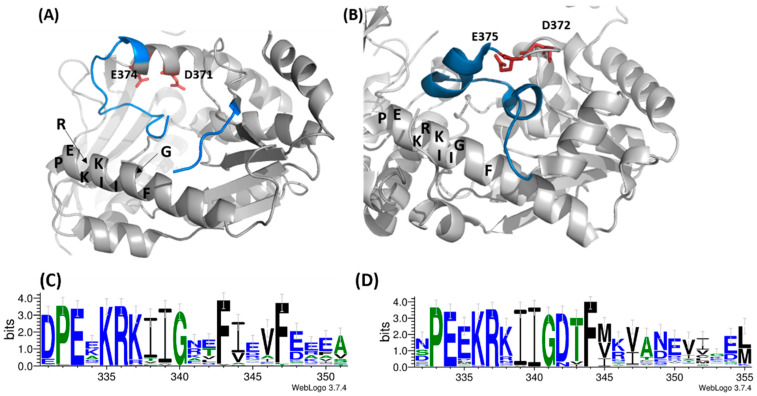
Close-up view of the ATPPase substrate binding pocket in (**A**) *P. falciparum* GMPS and (**B**) *H. sapiens* GMPS. The catalytic residues Asp371/Asp372 and Glu374/Glu375 in the two structures are shown as orange/red sticks, respectively, and the lid-loop in both structures is colored in blue. It can be observed that, whereas the loop enters the substrate binding pocket in the parasite enzyme, it remains outside the pocket in the mammalian counterpart. Residues of the signature motif PEXKRKIIGXXF on the helix facing the lid-loop are indicated. Weblogo of the same signature motif in (**C**) dimeric and (**D**) monomeric GMP synthetases. The selected regions include amino acid residues corresponding to stretch (**C**) Asp 331—Tyr 351 in the *P. falciparum* enzyme and (**D**) Ser 331—Met 355 in the human counterpart. The alignments were rendered using WebLogo 3.7.4. [41].

Upon rotation of the GATase domain, Cys113 is brought in close proximity to Cys377, as exemplified in the crystal structure of *Pf*GMPS_C89A/Gln (Figure 1). This latter cysteine takes part in the loop (lid-loop, residues 376–401) ensuing the helix, which holds the catalytic Asp371, a key residue in the AMP-XMP intermediate formation. This same helix also holds the catalytic Glu374 (invariant across all species), which, when mutated to Leu, results in an enzyme in which inter-domain cross-talk upon ATP- and XMP-binding to the ATPPase domain is abolished, and which is thought to be the key residue for allosteric activation of this enzyme. Indeed, these two catalytic residues are believed to participate in the positioning of NH_3_ in front of the adenyl-XMP bond for the attack to form GMP [10]. Once the 85° rotation has taken place, the so-called lid-loop 376-401 is deeply buried into the ATPPase domain (Figure 6A) and therefore also contributes, together with the dimer interface, to form a scaffold for the binding of the substrate and ultimately to displace the products of the reaction. The release of the lid-loop most probably triggers the rotation of the GATase domain back to its resting state, a hypothesis supported, among others, by the fact that this latter is not rotated in known crystal structures where the lid-loop is either disordered (all except *Hs*GMPS) or ordered but not present in the ATPPase pocket, as seen for *Hs*GMPS (Figure 6B).

With the aim of studying whether these observations are fortuitous, we performed a bioinformatics analysis to establish the extent to which the catalytic lid-loops are conserved within the two identified phylogenetic ensembles. These studies indicated that within each ensemble, there is an important conservation of both primary and secondary structures (Supplementary Figure S6), whereas they differ across ensembles with the exception of the IK(T/S)HHN motif. Indeed, this latter is essential for the synthesis of the adenyl-XMP intermediate through residues His388 and His389, as well as the formation of GMP through the interactions of Asn390 [11]. Despite the abovementioned differences, careful inspection reveals that in the vicinity of this loop there are conserved structural elements, such as the α-helix (D331—Y351 (*Pf*)/S332—M355 (*Hs*) in front of the helix bearing the catalytic residues, as well as the helix, which is the first structural element in the ATPPase domain (H241—Y251 (*Pf*)/T218—G235 (*Hs*), Figure 6A,B). While the latter helix does not show particular sequence conservation, the first displays a signature motif, which to the best of our knowledge has not been reported earlier and which corresponds to PEXKRKIIGXXF (Figure 6C,D). Interestingly, these two conserved α-helices appear more or less unwounded in the crystal structure of the double mutant (Supplementary Figure S7).

## 4. Discussion

The three-dimensional structure of the double mutant *Pf*GMPS_C89A_C113A indicated that a rotation of the GATase domain of approximately 170° and a ~1.5 Å translation has taken place, as compared to its position in the *Pf*GMPSwt structure. Whether this corresponds to a physiologically relevant form seems unlikely and is in any case uncertain. As opposed hereto, the earlier observed crystal structure of the single mutant *Pf*GMPS_C89A in complex with Gln, in which an 85° rotation had taken place upon the binding of glutamine, seems more realistic. Indeed, rotation of the GATase domain upon addition of glutamine has also been observed in a very recent study of *A. fumigatus* GMPS, for which the rotation was established by mass ion-mobility mass spectrometry, IM-MS [42]. By all means, besides presenting an enzyme with a partly destroyed substrate binding pocket in the ATPPase domain (Supplementary Figure S7), in *Pf*GMPS_C89A_C113A, the interactions that are critical for ensuring correct ammonia channeling from the GATase domain to the ATPPase domain are modified. It is tempting to think that there is a relation between this observation and the fact that upon rotation, in the native structure, Cys377 may form a disulfide bridge with Cys113, as earlier observed for the C89A mutant structure [10]. Hence, mutation of Cys113 to an alanine precludes the enzyme from resting in a conformation in which ammonia is in the correct position for attacking the AMP-XMP intermediate, as observed earlier in the 85° rotated enzyme (Supplementary Figure S8). In this sense, C113 could play the role of a barrier/manacle which prevents the enzyme from adopting a monomeric and partly unstructured and inactive form. This makes sense when recalling that C113 is present only in *P. falciparum*, *P. vivax* and *P. knowlesi*, and that the purine nucleotide metabolic pathway constitutes the sole source of purine nucleotides to the rapidly multiplying parasite. As concerns GMPS’ from the phylogenic ensemble containing monomeric enzymes, as revealed in this study, this issue is irrelevant since this group of enzymes with their extra-domain mimic the dimer-interface and therefore do not seem to be concerned by the destabilization of the ATPPase binding pocket. Indeed, in principle, they should always display the same structure.

The lid-loop, which has been shown to be essential for all the key steps in the catalytic process, including inter-domain communication, enhancement of the substrate affinity, formation of the ammonia channel, expulsion of the reaction products from the substrate binding pocket, etc. [10], appears to be conserved within the two respective ensembles both from a primary and secondary structure point of view. Comparison across the two ensembles, however, indicates that here, only the lid-loop IK(T/S)HHN motif is conserved. Despite this lower degree of conservation when comparing the two ensembles directly, one against the other, it appears that two α-helices are conserved in the immediate surroundings of the lid-loop, and that one of these also holds a signature motif PEXKRKIIGXXF. The tertiary structure conservation of these α-helices throughout the existing three-dimensional structures of GMPS, regardless of their origin, and their unwinding in the inactive double mutant discussed herein, support their importance for the catalytic process. Altogether, our results suggest that future studies should target the residues within the newly discovered signature motif found in one of the two conserved α-helices in order to attribute the role of each of these amino-acid residues in the catalytic reaction.

## Figures and Tables

**Table 1:** Data collection and refinement statistics.

	*Pf*GMPS_C89A_C113A
Data collection	
Beamline	ID30-A ESRF
Wavelength (Å)	0.87290
Resolution range (Å)	30.0–2.80
Space group	*C*2
Cell dimensions	
a, b, c (Å)	111.65, 64.54, 56.33
α, β, γ (°)	90.00, 90.03, 90.00
number of reflections	24,244
number of unique reflections	9228
R_meas_ (%)	11.8 (46.5)
CC ½	0.99 (0.74)
I/σ(I)	9.3 (3.6)
Multiplicity	2.6 (2.7)
Completeness (%)	92.3 (93.5)
number of molecules/asymm. unit	1
Refinement	
R/R_free_ ^1^ (%)	27.4/31.4
number of atoms	
protein	4070
water	10
Average B-factor (Å^2^)	48.7
protein	48.8
water	21.5
RMSD	
Bond lengths (Å)	0.005
Angles (°)	1.005
Ramachandran	
Favored (%)	76.3
Allowed (%)	18.5
Outliers (%)	5.2

^1^ R_free_, R_factor_ calculated from 5% of the data excluded from refinement.

## Data Availability

The data presented in this study are openly available in the RCSB Protein Databank at Rutgers under PDB-ID “7ZU9”. The rest of the data presented in this study are available upon reasonable request from the corresponding author.

## Supplementary Materials

The following supporting information can be downloaded at: https://www.mdpi.com/article/10.3390/biom12070871/s1, Figure S1: Stabilization of the dimer interface by amino acid residues that are less conserved across the GMPS’ from the studied species; Figure S2: Close-up on the dimer interface; Figure S3: Phylogenetic tree of GATase domains from GMP Synthetases; Figure S4: Phylogenetic tree of ATPPase domains from GMP Synthetases; Figure S5: Primary and secondary structure conservation of the extra-domain present in “ensemble A” GMP synthetases; Figure S6: Primary and secondary structure conservation of the catalytic lid-loop in dimeric and monomeric GMP synthetases; Figure S7: Close-up view on the ATPPase substrate binding pocket in the *P. falciparum* GMPS_C89A_C113A double mutant; Figure S8: Ammonia channeling in *P. falciparum* GMPS; Table S1: Protein sequences used to generate the phylogenetic tree of *Pf*GMPS homologs.
