# Supplementary material for: Tertiary and Quaternary Structure Organization in GMP Synthetases: Implications for Catalysis
Source: Biomolecules. 2022 Jun 23;12(7):871. doi: 10.3390/biom12070871 (PMC9312489; doi:10.3390/biom12070871)
Supplement: Supplementary file 1 [file biomolecules-12-00871-s001.zip › biomolecules-1744863-supplementary.pdf]

Tertiary and Quaternary Structure Organization in GMP Synthetases: Implications for Catalysis

Supplementary Materials

Supplementary Table S1. Protein sequences used to generate the phylogenetic tree of *Pf*GMPS homologs. Organisms with an asterisk are from Yanai *et al*, 2002 [25].

Three letters code	Name	Group	Uniprot	Accession number
Aae*	*Aquifex aeolicus*	Aquificae		AAC06558.1
Ssp*	*Synechocystis sp.*	Cyanobacteria		BAL36329.1
Dra*	*Deinococcus radiodurans*	Deinococcus		AAF11428.1
Tth	*Thermus thermophilus*	Deinococcus	Q5SI28	
Aha*	*Alkalihalobacillus halodurans*	Firmicutes	Q9KF78	
Cbu	*Coxiella burnetii*	Proteobacteria	Q83BZ6	
Cje*	*Campylobacter jejuni*	Proteobacteria		CAL35363.1
Eco	*Escherichia coli*	Proteobacteria	P04079	
Hin	*Haemophilus influenzae*	Proteobacteria		AAC21891.1
Hpy*	*Helicobacter pylori*	Proteobacteria		ERA59126.1
Ngo	*Neisseria gonorrhoeae*	Proteobacteria	B4RJH7	
Nme*	*Neisseria meningitidis*	Proteobacteria		AAF42250.1
Pae*	*Pseudomonas aeruginosa*	Proteobacteria		AAG07156.1
Vch*	*Vibrio cholerae*	Proteobacteria		AAF93933.1
Xfa*	*Xylella fastidiosa*	Proteobacteria		AAF85228.1
Bga*	*Borrelia garinii*	Spirochaetes		AAT93750.1
Tma*	*Thermotoga maritima*	Thermotogae		AAD36883.1
Ape	*Aeropyrum pernix*	Archaebacteria	Q9Y933	
Mja	*Methanocaldococcus jannaschii*	Archaebacteria		
Pae	*Pyrobaculum aerophilum*	Archaebacteria	Q8ZT92	
Pho	*Pyrococcus horikoshii*	Archaebacteria		
Tar	*Thermococci archaeon*	Archaebacteria		RLF89122.1 + RLF89120.1
Dre	*Danio rerio*	Actinopterygii	B8JLW8	
Psp	*Polyodon spathula*	Actinopterygii		MBN3282960.1
Aca	*Acanthamoeba castellanii*	Amoebozoa		XP_004337602.1
Ddi	*Dictyostelium discoideum*	Amoebozoa		XP_640534.1
Xla	*Xenopus laevis*	Amphibia	Q5XG45	
Cte	*Capitella teleta*	Annelida	R7TZP7	
Ofu	*Owenia fusiformis*	Annelida		CAC9662317.1
Cci	*Cardiosporidium cionae*	Apicomplexa		KAF8822715.1

Gni	*Gregarina niphandrodes*	Apicomplexa		XP_011132670.1
Pfa	*Plasmodium falciparum*	Apicomplexa		XP_001347408.1
Tgo	*Toxoplasma gondii*	Apicomplexa		CEL74772.1
Ttr	*Thecamonas trahens*	Apusozoa		XP_013762482.1
Ool	*Orbilia oligospora*	Ascomycota		KAF3146082.1
Sce	*Saccharomyces cerevisiae*	Ascomycota	P38625	
Csp	*Ceratobasidium sp.*	Basidiomycota		KAG9097502.1
Ama	*Allomyces macrogynus*	Blastocladiomycotina		KNE63377.1
Bfl	*Branchiostoma floridae*	Cephalochordata		XP_035672724.1
Isc	*Ixodes scapularis*	Chelicerata	B7PKN7	
Nst	*Nymphon striatum*	Chelicerata		KAG1661047.1
Tcl	*Trichonephila clavata*	Chelicerata		GFR24954.1
Cre	*Chlamydomonas reinhardtii*	Chlorophyta	Q84UB2	
Ssp	*Scenedesmus sp.*	Chlorophyta		KAF8070969.1
Tsp	*Trebouxia sp.*	Chlorophyta		KAA6423168.1
Sro	*Salpingoeca rosetta*	Choanoflagellida		XP_004998503.1
Sen	*Synchytrium endobioticum*	Chytridiomycota		TPX33674.1
Pda	*Pocillopora damicornis*	Cniadaria		XP_027053323.1
Lch	*Latimeria chalumnae*	Coelacanthimorpha		XP_014345852.1
Aam	*Amphibalanus amphitrite*	Crustacea		XP_043219697.1
Ana	*Armadillidium nasatum*	Crustacea	A0A5N5SXI2	
Pva	*Penaeus vannamei*	Crustacea		XP_027227701.1
Gth	*Guillardia theta*	Cryptophyta		XP_005836417.1
Smi	*Symbiodinium microadriaticum*	Dinoflagellata		CAE7898894.1
Aja	*Anneissia japonica*	Echinodermata		XP_033113703.1
Spu	*Strongylocentrotus purpuratus*	Echinodermata	A0A7M7NWA3	
Ldo	*Leishmania donovani*	Euglenoza		CAC5429972.1
Tcr	*Trypanosoma cruzi*	Euglenoza		ESS64926.1
Cto	*Chrysochromulina tobinii*	Haptophyta		KOO23560.1
Ehu	*Emiliania huxleyi*	Haptophyta		XP_005760952.1
Cci	*Cephus cinctus*	Hexapoda		XP_015604604.1
Cfe	*Ctenocephalides felis*	Hexapoda		XP_026473819.1
Cma	*Callosobruchus maculatus*	Hexapoda	A0A653D5E0	
Dme	*Drosophila melanogaster*	Hexapoda	Q9VIE7	
Hsa	*Homo sapiens*	Mammalia	P49915	
Mmu	*Mus musculus*	Mammalia	Q3THK7	
Ovu	*Octopus vulgaris*	Mollusca	A0A7E6FE49	
Sph	*Sepia pharaonis*	Mollusca		CAE1294826.1
Jfl	*Jimgerdemannia flammicorona*	Mucoromycotina		RUS26741.1
Cel	*Caenorhabditis elegans*	Nematoda	Q09580	
Nam	*Necator americanus*	Nematoda		XP_013297685.1
Tps	*Trichinella pseudospiralis*	Nematoda		KRY86210.1
Hme	*Histomonas meleagridis*	Parabasalidea		KAH0789514.1

Pma	*Petromyzon marinus*	Petromyzontida		XP_032818278.1
Tsp	*Trichoplax sp.*	Placozoa		RDD44187.1
Csi	*Clonorchis sinensis*	Platyhelminthes		KAG5449599.1
Egr	*Echinococcus granulosus*	Platyhelminthes		XP_024347903.1
Cme	*Cyanidioschyzon merolae*	Rhodophyta		XP_005536805.1
Gga	*Gallus gallus*	Sauropsida	F1NAQ8	
Oja	*Oxyura jamaicensis*	Sauropsida		XP_035190688.1
Ath	*Arabidopsis thaliana*	Streptophyta	Q9CAD1	
Oeu	*Olea europaea*	Streptophyta		CAA3026992.1
Ppa	*Physcomitrium patens*	Streptophyta	A9RGM9	
Ptr	*Populus trichocarpa*	Streptophyta	B9GWS1	
Hdu	*Hypsibius dujardini*	Tardigrada		OQV18758.1
Cin	*Ciona intestinalis*	Urochordata		XP_026689623.1
Smu	*Smittium mucronatum*	Zoopagomycota		OLY82375.1

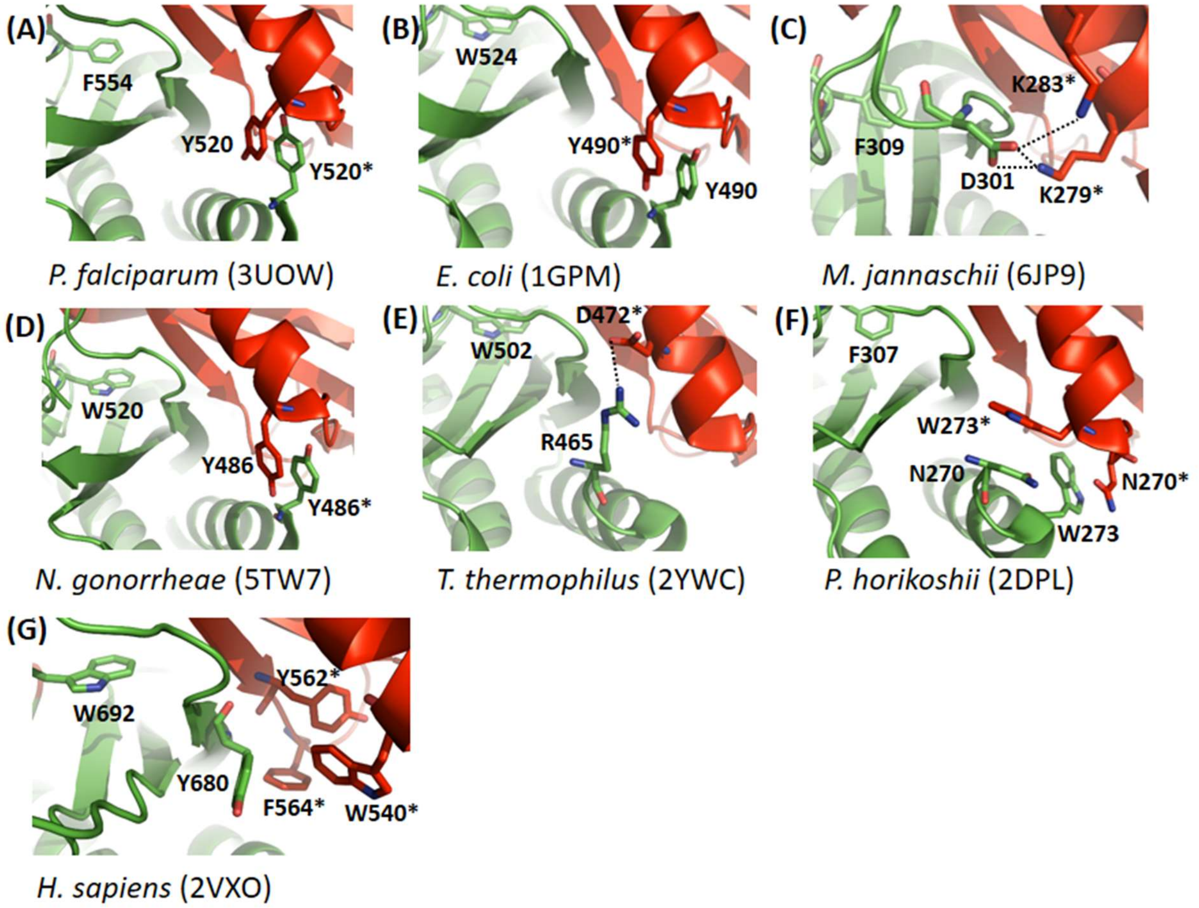

Supplementary Figure S1. Stabilization of the dimer interface by amino acid residues that are less conserved across the GMPS' from the studied species. Stippled lines indicate salt-bridges. For GMPS from (**A**) *P. falciparum*, (**B**) *E. coli*, (**C**) *M. jannaschii*, (**D**) *N. gonorrhea*, (**E**) *T. thermophilus* and (**F**) *P. horikoshii*, residues from monomer A are shown in green and monomer B in red with the interacting residues from monomer B marked by an asterisk. For *H. sapiens* GMPS (**G**) all residues are from the same polypeptide chain, with the so-called D1 extra-domain depicted in red, and residues from the conventional ATPPase domain colored in green, and mimes the dimer interface as seen for the above-mentioned enzymes. Interacting amino-acid residues from this extra-domain are labelled with asterisks.

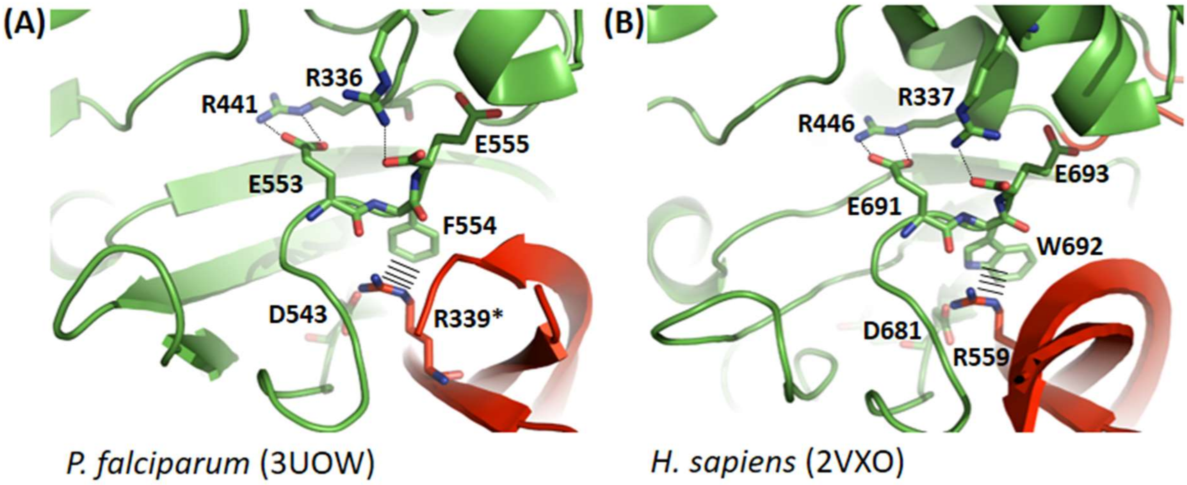

Supplementary Figure S2. Close-up on the dimer interface as formed in (**A**) *P. falciparum* GMPS and the corresponding region in (**B**) *H. sapiens* GMPS. Lines indicate π – cation interactions. For *Pf*GMPS, residues from monomer A are shown in green and monomer B in red with the interacting residues from monomer B marked by an asterisk. In *Hs*GMPS all residues are from the same polypeptide chain, and the so-called D1 extra-domain is colored in red, and residues from the conventional ATPPase domain are colored in green and mimes the dimer interface as seen for the above-mentioned enzymes (Supplementary Figure S1 A-F) and exemplified here in *P. falciparum* GMPS. It can be observed that all the interacting amino acid residues in this area are strictly conserved between the enzymes from the two species, with the exception of Phe554 (*Pf*)/Trp692 (*Hs*) which are a part of the C-terminal signature motif. It should be highlighted that Arg336 (*Pf*) and Arg337 (*Hs*) interact with the C-terminus in both enzymes.

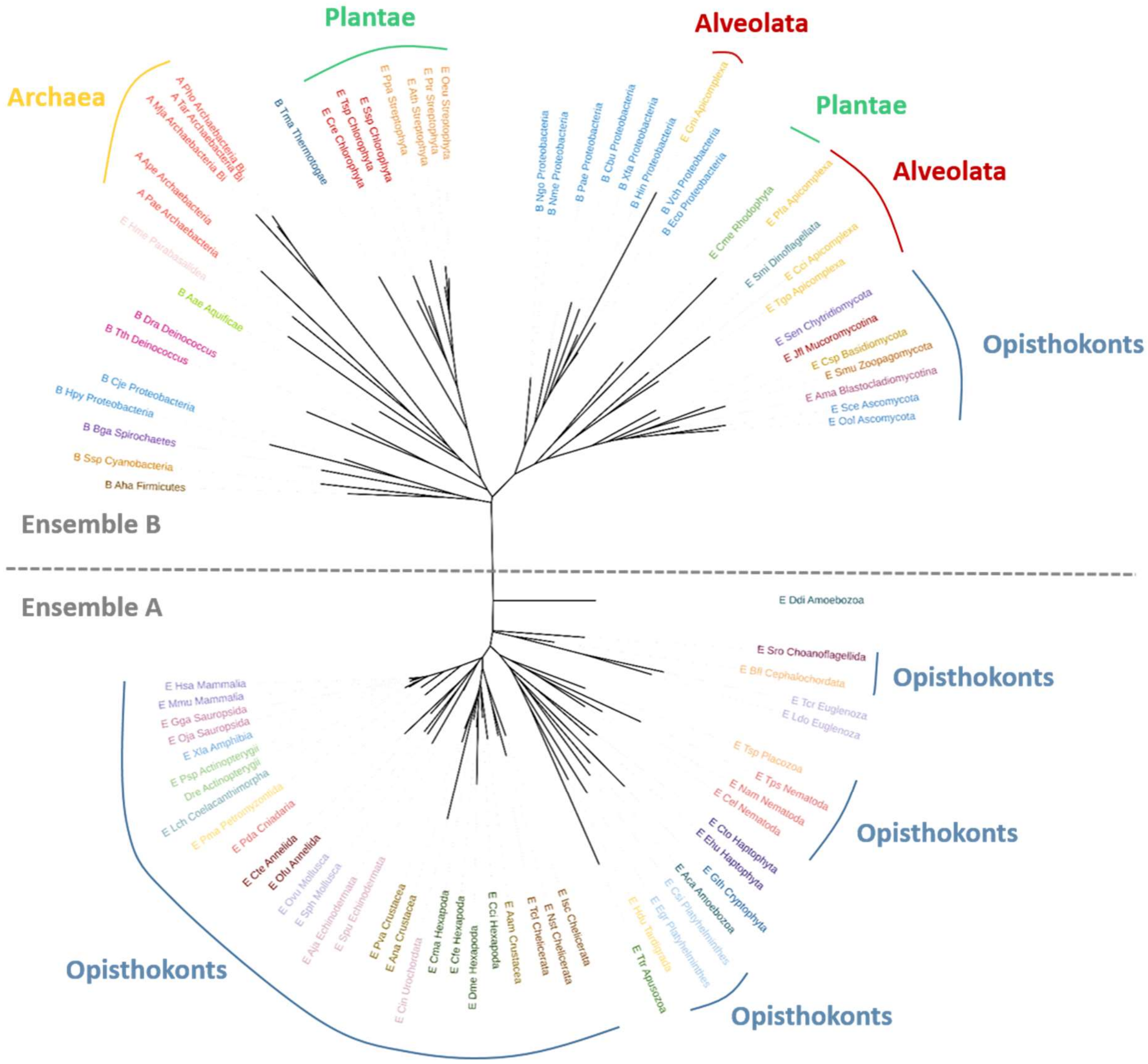

Supplementary Figure S3. Phylogenetic tree of GATase domains from GMP Synthetases. A Maximum Likelihood phylogeny based on 158 amino acids positions of 87 sequences was constructed with PhyML [30] using GMPS from eukaryotes, archaea and bacteria.

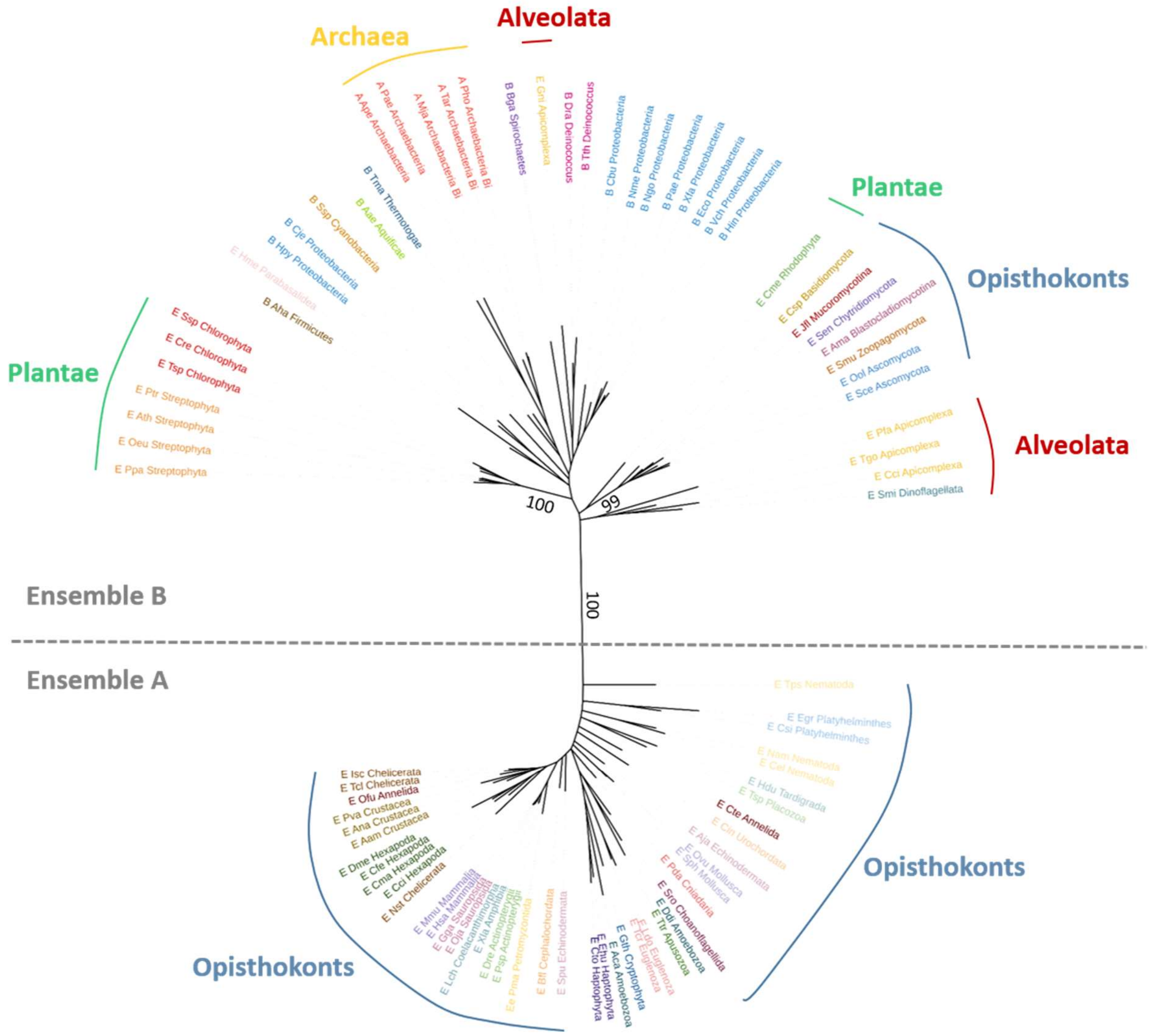

Supplementary Figure S4. Phylogenetic tree of ATPPase domains from GMP Synthetases. A Maximum Likelihood phylogeny based on 152 amino acids positions of 87 sequences was constructed with PhyML [30] using GMPS from eukaryotes, archaea and bacteria.

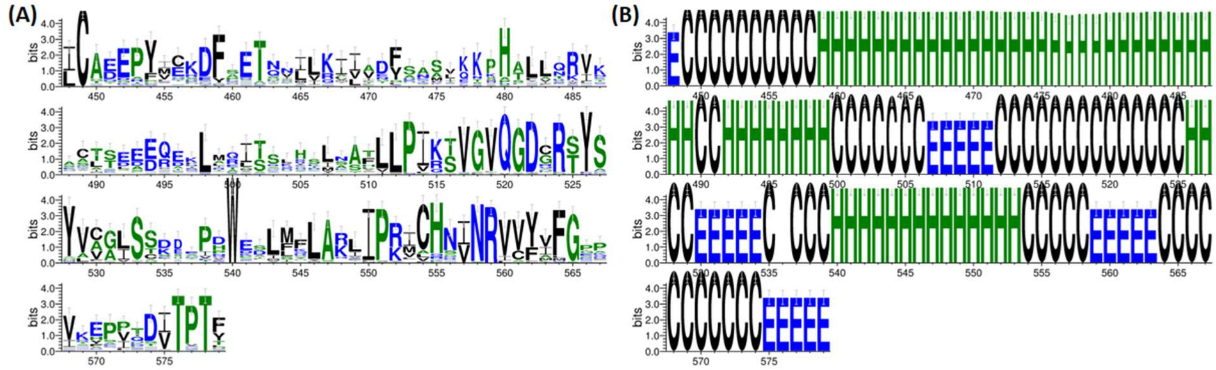

Supplementary Figure S5. (**A**) Primary- and (**B**) secondary structure conservation of the extra-domain present in “ensemble A” GMP synthetases. The secondary structure predictions by PSIPRED [35] for the 46 aligned sequences are shown as a sequence logo with H being “helix”, C being “coil” and E “beta-strand”. The selected region includes amino-acid residues corresponding to the stretch Ile448 - Phe579 in the human enzyme. Alignments have been rendered using WebLogo 3.7.4. [41].

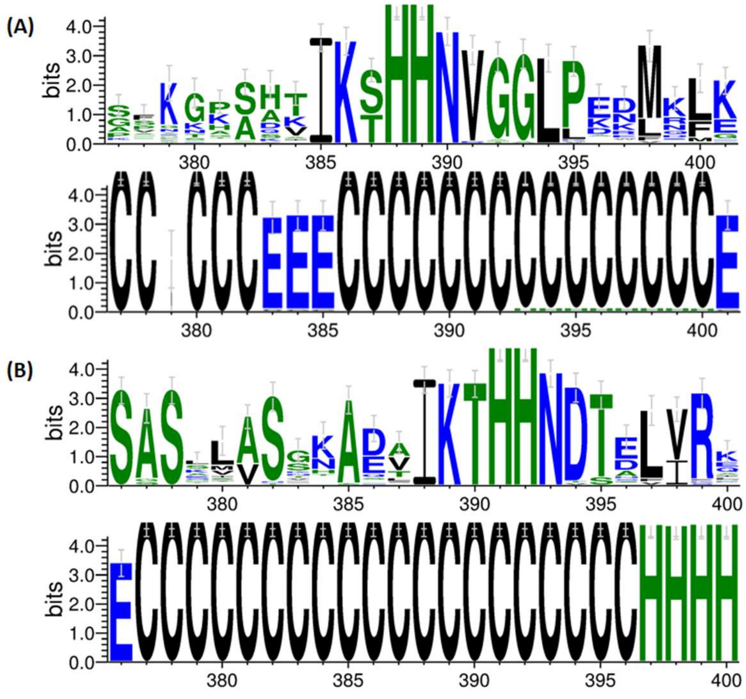

Supplementary Figure S6. Primary- and secondary structure conservation of the catalytic lid-loop in (**A**) dimeric- and (**B**) monomeric GMP synthetases. The secondary structure predictions by PSIPRED [35] for the aligned sequences (44 for dimeric and 46 for monomeric GMP synthetases) are shown as a sequence logo on the bottom line with H being “helix”, C being “coil” and E “beta-strand”. The selected regions include amino-acid residues corresponding to the stretch (**A**) Cys377 - Lys401 in the *P. falciparum* enzyme, and (B) Ser376 - Asp394 in the human counterpart. The alignments have been rendered using WebLogo 3.7.4. [41].

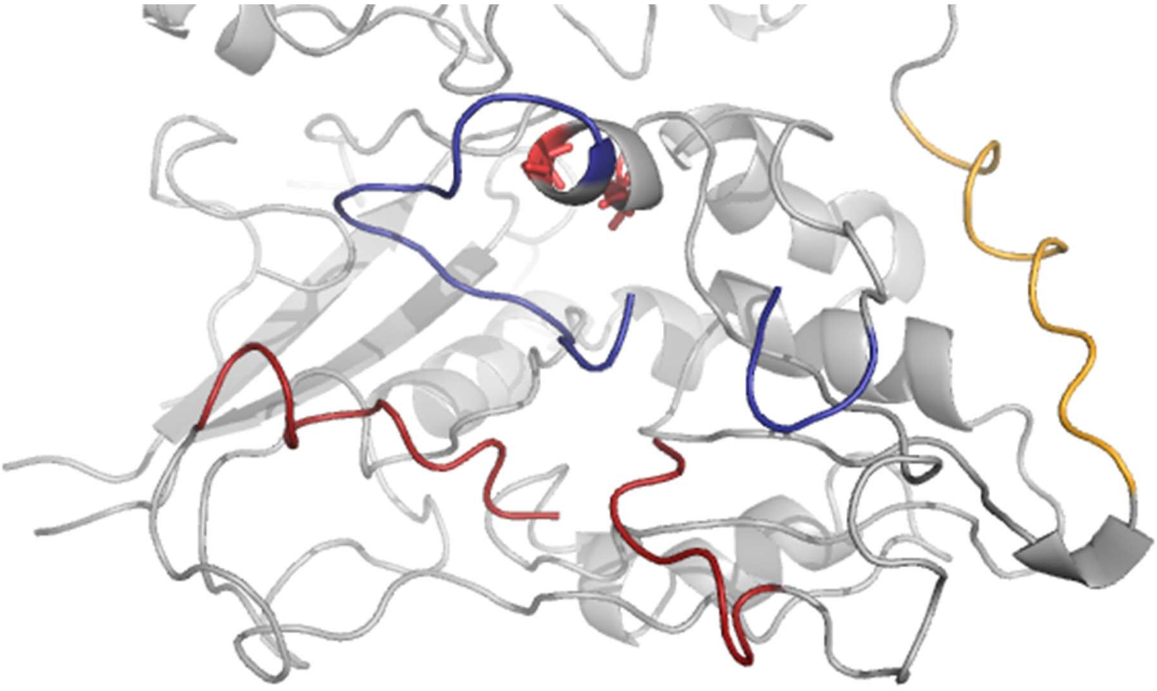

Supplementary Figure S7. Close-up view on the ATPPase substrate binding pocket in the *P. falciparum* GMPS_C89A_C113A double mutant. The catalytic residues, Asp371 and Glu374 are shown as red sticks, and the lid-loop is colored in blue. It can be observed that the two α-helices which are structurally conserved among all the existing three-dimensional structures are in the process of being unwound. The α-helix which contains the signature motif "PEXKRKIIGXXF" is colored in firebrick red, whereas the α-helix which initiates the ATPPase domain is colored in light orange. The orientation is similar to that given in Figure 6.

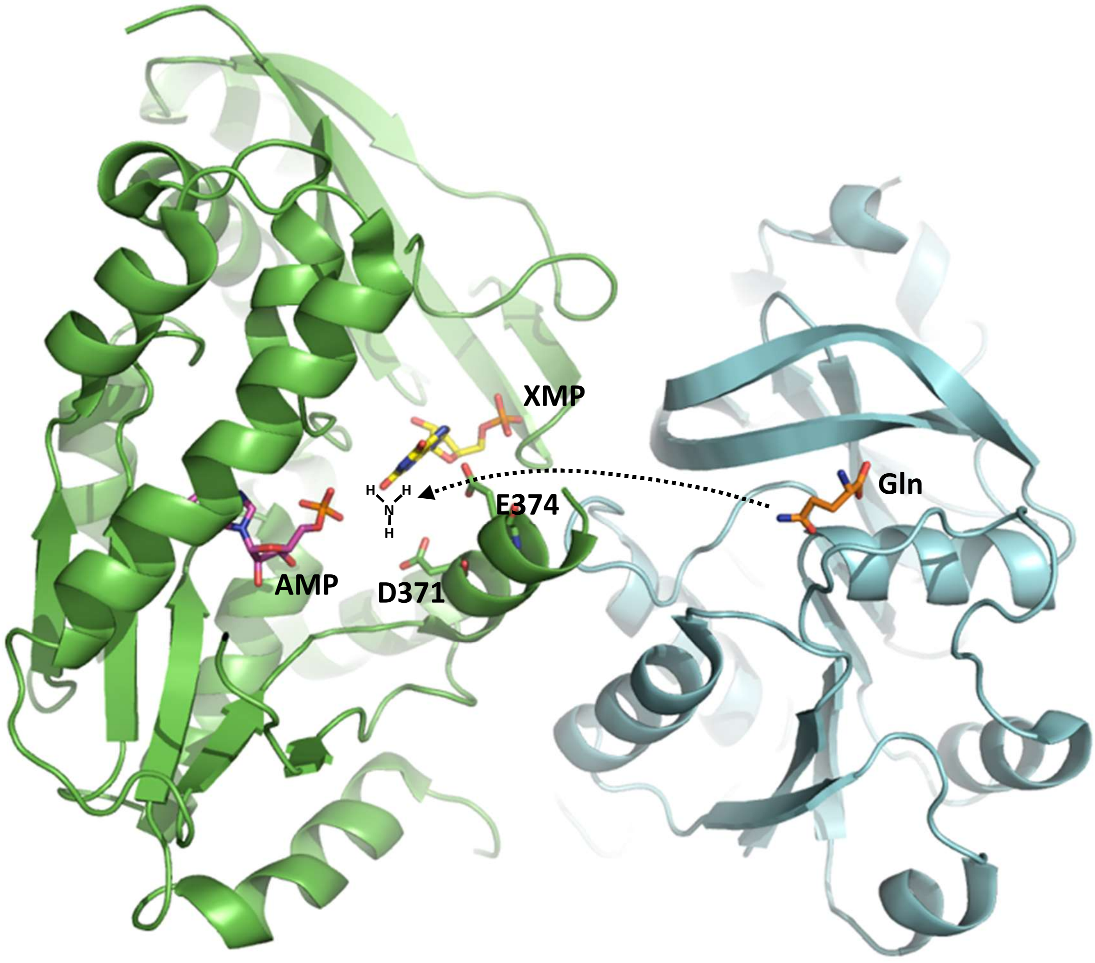

Supplementary Figure S8. Ammonia channeling in *P. falciparum* GMPS from the GATase domain to the ATPPase domain is possible when GATase and ATPPase domains are positioned in their respective orientations as observed in the crystal structure of the GMPS_C89A mutant [10]. The catalytic residues, Asp371 and Glu374 of the ATPPase domain are shown as green sticks, and XMP and AMP are shown in yellow and orange, respectively. Gln as situated in the active site of the GATase domain is depicted in orange.
